# 189. Potential Tiger-to-Human Transmission of SARS-CoV-2 at a Tennessee Zoo: A One Health Approach to Outbreak Investigation

**DOI:** 10.1093/ofid/ofab466.189

**Published:** 2021-12-04

**Authors:** Heather N Grome, Becky Meyer, Erin Read, Martha Buchanan, Andrew C Cushing, Kara J Levinson, Linda S Thomas, Zachary Perry, Krista Queen, Anna Uehara, Suxiang Tong, Ying Tao, Mary-Margaret A Fill, Timothy F Jones, William Schaffner, John R Dunn

**Affiliations:** 1 Centers for Disease Control and Prevention, Nashville, TN; 2 Knox County Health Department, Knoxville, Tennessee; 3 University of Tennessee, Knoxville, Tennessee; 4 Division of Laboratory Services, TN Department of Health, Nashville, Tennessee; 5 Division of Viral Diseases, Centers for Disease Control and Prevention (CDC), Atlanta, Georgia; 6 Centers for Disease Control and Prevention (CDC), Atlanta, Georgia; 7 Tennessee Department of Health, Nashville, Tennessee; 8 Vanderbilt University Medical Center, Nashville, Tennessee

## Abstract

**Background:**

Human-to-feline and airborne transmission among cats of Severe Acute Respiratory Syndrome Coronavirus-2 (SARS-CoV-2) has been described, though documented feline-to-human transmission has not been reported. In October 2020, all 3 Malayan tigers at a Tennessee AZA accredited zoo were diagnosed with symptomatic SARS-CoV-2 infection. We investigated to determine source and prevent further transmission.

**Methods:**

Tiger nasal swab specimens were tested at the National Veterinary Services Laboratories (NVSL). An environmental assessment at the zoo was completed. We interviewed 18 staff who interacted with the tigers during the 2 weeks before animal symptom onset. Confirmed human cases were defined as persons testing positive for SARS-CoV-2 by RT-PCR during September 28–October 29, with tiger interaction during their 14-day incubation period. Interviewed staff had repeat SARS-CoV-2 RT-PCR and serum IgG testing on October 29. Tigers and staff testing positive had specimens sent to CDC for genomic sequencing. Tiger sequences were compared phylogenetically with 30 geographically associated human cases collected within 2 weeks of the outbreak and > 200 background sequences from TN.

**Results:**

NVSL confirmed SARS-CoV-2 infection in all 3 tigers. Environmental assessment identified fencing between humans and animals allowing airflow and an open outdoor exhibit observation point above the habitat. Confirmed cases were identified in a tiger keeper and veterinary assistant; both developed symptoms after exposure to symptomatic tigers and one sample was genotyped. Staff did not report known contact with ill visitors. All staff were negative for SARS-CoV-2 IgG. The tigers and most temporally and geographically associated cases had genetic sequences in clade 20G and B.1.2. Tiger sequences were 3-6 single nucleotide polymorphisms different from the positive tiger keeper (Figure).

Figure. Whole-genome phylogenetic analysis.

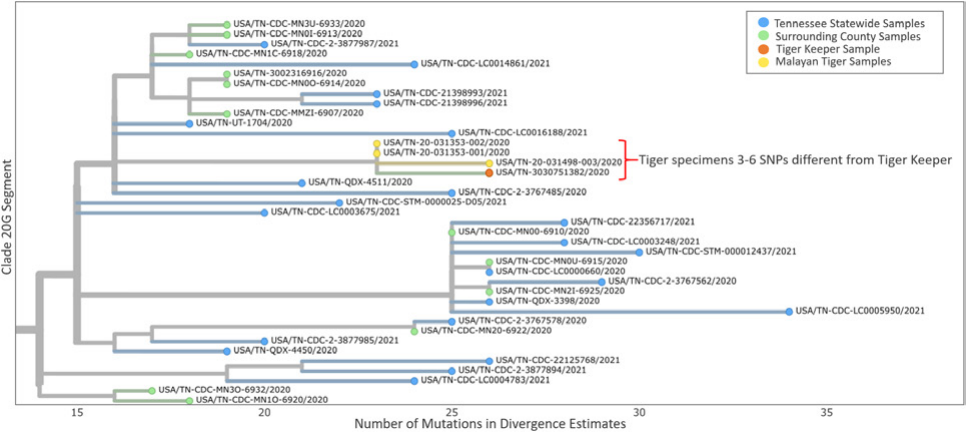

Whole-genome phylogenetic analysis from a portion of clade 20G showing divergence estimates from SARS-CoV-2 Wuhan-Hu-1 reference genome with sequences from humans living in Tennessee and Malayan tigers sampled during the outbreak investigation in October 2020. Sequence analysis showed 3-6 single nucleotide polymorphisms (SNPs) differences between one human tiger keeper and all three tiger sequences. Differences are indicated by one-step edges (lines) between colored dots (individual SARS-CoV-2 sequenced infections). Numbers indicate unique sequences. Note not all analyzed sequences are shown in this figure.

**Conclusion:**

Using a One Health approach, we concluded the index tiger was likely infected via transmission from an ill visitor at an exhibit observation point or unidentified asymptomatic staff. Infection spread to the other 2 tigers and tiger-to-human transmission to 2 staff is possible thereafter. The zoo was advised on infection control practices for humans and animals, and no additional cases were identified.

**Disclosures:**

**William Schaffner, MD**, **VBI Vaccines** (Consultant)

